# Interplay between Oxidative Stress, Inflammation, and Amyloidosis in the Anterior Segment of the Eye; Its Pathological Implications

**DOI:** 10.1155/2020/6286105

**Published:** 2020-06-03

**Authors:** Roxanna Perez-Garmendia, Alicia Lopez de Eguileta Rodriguez, Ivan Ramos-Martinez, Nayeli Martínez Zuñiga, Roberto Gonzalez-Salinas, Hugo Quiroz-Mercado, Edgar Zenteno, Eleazar Ramírez Hernández, Luis Fernando Hernández-Zimbrón

**Affiliations:** ^1^Research Department, Asociación para Evitar la Ceguera, IAP, México City 04030, Mexico; ^2^Hospital Universitario Marqués de Valdecilla Santander, Spain; ^3^Laboratorio Nacional de Óptica de la Visión, Gto, León, Mexico; ^4^Biochemistry Department, Facultad de Medicina, Universidad Nacional Autónoma de México, Ciudad de México, Mexico

## Abstract

There are different pathologies associated with amyloidogenic processes caused by the increase of reactive oxygen species (ROS) and the overactivation of inflammatory responses. These alterations are present in different regions of the anterior segment of the eye, and they have been associated with the development and progression of ocular pathologies, such as glaucoma, dry eye syndrome, keratitis, and cataracts among other pathologies. *Aim*. To discuss briefly the anatomical characteristics of the anterior segment of the eye and describe the interaction between oxidative stress (OS) and inflammatory responses, emphasizing the misfolding of several proteins leading to amyloidogenic processes occurring in the anterior segment and their implications in the development of ocular diseases. We performed a search on PubMed, CINAHL, and Embase using the MeSH terms “eye,” “anterior segment”, “inflammation”, “oxidative stress”, and “amyloidosis”. The search encompassed manuscripts published up to April 2019. A hundred forty-four published studies met the inclusion criteria. We present the current knowledge regarding the interaction between OS and the activation of inflammatory processes and how both can cause conformational changes in several peptides and proteins in each compartment of the anterior segment. However, we found that there is no consensus about which factor is the first to cause amyloidosis. Our conclusions suggest that there is an interplay among these factors forming a vicious cycle that leads to the loss of protein structure in ocular pathologies, and multifactorial therapies should be developed to avoid protein misfolding and to stop the progression of ocular pathologies.

## 1. Introduction

The eye is highly exposed to light, robust metabolic activity, and, in certain regions, high oxygen tension, is particularly susceptible to oxidative damage. There are different pathologies associated to an increase of reactive oxygen species (ROS) that causes oxidative stress (OS), activation of inflammatory responses, and both of them could induce amyloidosis processes. These altered mechanisms are present in different regions of the anterior segment of the eye and are associated with the development and progression of glaucoma, dry eye syndrome, keratitis, and cataracts, among other pathologies.

The anterior segment of the eye comprises all the structures lying between the ocular surface of the corneal epithelium and the posterior capsule of the lens. Here, we will discuss briefly the anatomical characteristics of the anterior segment, followed by a description of how the anterior segment is affected by the interplay between OS and amyloidosis and their implications in the development and progression of ocular pathologies.

OS is a process that overwhelms the antioxidant defenses of the cells through the generation of reactive oxygen species (ROS). This may be either due to an overproduction of ROS or to a failure of the cell buffering mechanisms [1]. ROS include superoxide anions and play an essential role in cell signaling and regulation. They are generated as a by-product of oxidative metabolism, in which energy activation and electron reduction are involved; however, protein and DNA oxidation and lipid peroxidation are consequences of OS due to imbalance that occurs at a molecular/cellular level when free radical production exceeds antioxidant scavenging capacity [1, 2].

**Figure 1:**
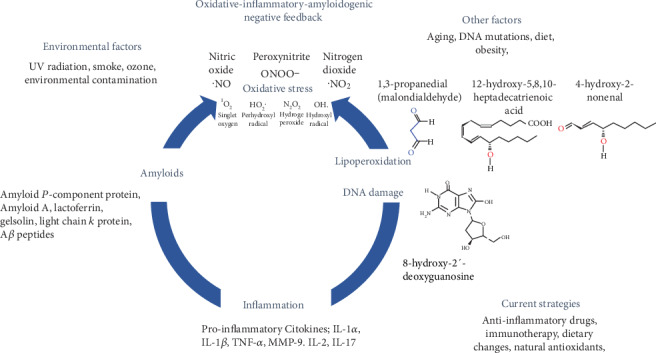
Schematic representation of the oxidative-inflammatory-amyloidogenic negative feedback that occurs in the anterior segment of the eye. The eye is one of the major targets of the OS attack, inflammation, and amyloidogenic processes due to exposition on several environmental factors that contributes to the development and progression of ocular diseases, such as glaucoma, dry eye syndrome, keratitis, and cataracts among other pathologies. The three main factors are involved in a vicious circle that we called “oxidative-inflammatory-amyloidogenic negative feedback.”

The chemical reactivity of ROS varies from the very toxic hydroxyl (˙OH) to the less reactive superoxide radical (O_2_˙ ¯). The initial product, O_2_˙ ¯, result from the addition of a single electron to molecular oxygen. O_2_˙ ¯ is rapidly dismutated by superoxide dismutase (SOD), yielding H_2_O_2_ and O_2_, which can be reused to generate superoxide radical. In the presence of reduced transition metals (iron and cooper), H_2_O_2_ although less reactive that O_2_˙ ¯ and highly diffusible can be converted into the highly reactive ˙OH. The detoxification is achieved through the involvement of several enzymatic and nonenzymic antioxidant mechanisms that protect the tissues against OS which are available to the cells in different cellular compartments [1–4] (Figure 1).

The major sources of ROS include mitochondrial respiratory chain, xanthine/xanthine oxidase, myeloperoxidase, uncontrolled arachidonic acid (ARA) cascade, and activation of NADPH oxidase. These reducing systems use electron donors such as glutathione (GSH), NADPH, NADH, FADH2, and thioredoxin. The main reducing system in the eye is the glutathione system that includes reduced GSH, oxidized glutathione (GSSG), and a number of related enzymes [5–7]. Glutathione peroxidase reduces H_2_O_2_ to water and leads to the oxidation of GSH to GSSG. The reduced state of GSH is maintained by glutathione reductase in which NADPH is needed, hence the importance of glucose-6-phosphate dehydrogenase as well. This system is capable of detoxifying H_2_O_2_, dehydroascorbic acid, and lipid peroxides and maintains protein thiols in a reduced state. Other reducing agents include thioltransferase that reduces protein thiols by using reduced GSH [7] and thioredoxin that uses NADPH to maintain mitochondrial proteins in a reduced state [8]. Antioxidant enzymes contribute to the protective role against ROS, like superoxide dismutase, especially SOD2 that converts O_2_˙ ¯ to hydrogen peroxide.

Excess free radical production disturbs redox status and can modulate leading the expression of a variety of immune and inflammatory molecules leading to inflammatory processes, exacerbating inflammation, and affecting tissue damage age. The primary target of ROS in lipids in the cell membrane which undergo increased lipid peroxidation (LPO), DNA oxidation, protein carbonyl formation, thereby impairing cell structure and function.

Inflammation is normally defined as a response to the stimulation caused by invading pathogens or endogenous signals, such as damaged cells and/or OS, that result in tissue repair or different pathologies, attributed to an uncontrolled response. It is difficult to understand all the mechanisms, context, and the role of inflammation during physiological and nonphysiological immune responses. Each tissue in the body evokes distinct characteristics of inflammation as a result of general and local molecular, immunological, and physiological processes. If we talk about physiological parameters of a protective immune response, inflammation is absolutely necessary for efficient immunity, including tissue healing and return to homeostasis, and physiological inflammation is self-limiting and self-regulated [9].

Amyloidosis is protein-misfolding diseases characterized by the deposition of aggregated protein in the form of abnormal fibrils. The fibrils are composed of protein that often has been mutated, partially fragmented, or otherwise altered, which predisposed them to adopt an abnormal conformation and are deposited in tissues as abnormal insoluble fibrils that cause structural and functional disruptions [9–11]. OS is strongly associated with an inflammatory response, as a cause or consequence. However, whether both of them are related to an amyloidosis process in the anterior segment of the eye remains to be determined. In this review, we revise ophthalmological conditions potentially involving reactive oxygen species (ROS), inflammation, and amyloidosis considering its interactions in diseases of the human anterior segment.

## 2. Methods

This review focuses on published articles that address the subject of the aging process in the anterior segment of the eye and the associated molecular and physiological events. We performed a search in PubMed, CINAHL, and Embase for the published literature available using the MeSH terms “eye,” “anterior segment”, “inflammation”, “oxidative stress”, and “amyloidosis”. We generated searches to account for synonyms of these keywords and MESH headings, as follows: (1) “Eye” AND “inflammation” OR “anterior segment amyloidosis” and (2) “Anterior segment” AND “oxidative stress” OR “anterior segment” AND “molecular changes”. We used no language restrictions. The search encompassed manuscripts published up to January 2020. Abstracts from meetings were not included, as they usually do not contain enough information to perform a proper evaluation. We identified 144 published studies that met the inclusion criteria.

## 3. Results

### 3.1. Ocular Surface and Cornea

The lacrimal functional unit, defined as the ocular surface, lacrimal gland, and their neural interconnectivity, maintains ocular surface homeostasis, and disruption of this leads to tear film instability. Meibomian glands are responsible for the oily component of the tear film, becoming dysfunctional in most patients aged 60 and older, causing rapid evaporation of the tear, an increase of free radical with subsequent dry eye symptoms, discomfort, and visual disturbances [2, 11, 12].

It has been demonstrated that tears present a powerful content of antioxidant enzymes like superoxide dismutase-1 (SOD-1), glutathione peroxidase (GPx), and nonenzymatic antioxidants or antioxidant precursors; glutathione (GSH); uric acid (UA) cysteine (cys) and tyrosine and ascorbate. Besides, high concentrations of lactoferrin in tears help to reduce the ·OH formation by chelation of redox-active iron that could be formed by iron—assisted Fenton Chemistry. However, these antioxidants are not enough to inhibit the consequent inflammatory response and the progression of dry eye.

Inflammatory changes in the ocular surface related to a chronic oxidative stress state. In earlier stage of the inflammatory cycle, ocular surface damage leads to a compensatory reflex stimulation of the gland. In experimental studies, excessive reflex stimulation of the lacrimal gland may induce a neurogenic inflammatory cytokine response within the gland, leading to the release of inflammatory markers into the tear film [12].

This inflammatory setting prompts the maturation of antigen-presenting cells (APCs) and resident dendritic cells in the ocular tissue. The specific initiating the mature APCs bearing self-antigens travel to regional lymph nodes through the afferent lymphatic vessels, where they prime naive T cells, which subsequently transform into the CD4^+^ T-helper cell subsets T_H_1 and T_H_17. These subsets are effector cells that migrate through the efferent vasculature to the ocular surface, where they are believed to induce epithelial damage via cytokine release [12]. There are previous reports indicating that dry eye-related ocular surface inflammation is mediated by lymphocytes and conjunctival inflammation represented by T cell infiltrates and overexpression of CD3, CD4, and CD8; lymphocyte activation markers CD11a and HLA-DR and proinflammatory cytokines, like interleukin IL-1*β*, IL-6, IL-8, TNF-alpha, and matrix metalloproteinases (MMPs) mainly MMP-9 are also involved in the pathogenesis of dry eye [13]. Tear film hyperosmolarity, tear film instability, and inflammation go hand in hand: tear film hyperosmolarity leads to ocular surface epithelial cell hyperosmolarity, which stimulates the inflammatory cascade involving mitogen-activated protein kinases and nuclear factor *κ*-light-chain-enhancer of activated B cells signaling pathways, cytokines (interleukin [IL] 1*α*, IL-1*β*, tumor necrosis factor [TNF] *α*, and MMP-9. T-cell recruitment and subsequent secretion of additional cytokines scale the cycle of inflammation [14].

The T-cell subsets T_H_1 and T_H_17 migrate to the ocular surface, where additional inflammatory mediators are released, including interferon *γ*, TNF-*α*, IL-2, and IL-17. Interferon *γ* is linked to goblet cell dysfunction and death by altering mucin. Murine experiments have shown T_H_17 cells and their resultant IL-17 cytokines to be dominant players in the disruption of corneal barrier function. Interleukin 17, along with IL-1 and TNF-*α*, seems to stimulate the release of MMPs by the corneal epithelium, which disrupts corneal epithelial tight junctions. In addition, IL-17 upregulates expression of vascular endothelial grown factors C and D that promotes corneal lymphangiogenesis in dry eye diseases [12–14].

These inflammatory changes, also induce an exacerbated production of free radicals, and all together affects the cornea that suffers changes in its shape and optical properties, including corneal steepening, measurable by keratometry, and a shift in toxicity from with-the-rule to against the-rule astigmatism and increased collagen interfibrillar spacing, as well as an increased thickness of Descemet' s membrane [15].

The cornea is a transparent avascular tissue covering the front portion of the eye comprised of five layers: the corneal epithelium, Bowman's layer, the corneal stroma, Descemet's membrane, and the endothelium [16]. It is also richly supplied by sensory nerve fibers with a central corneal nerve density of proximately 7000 nociceptors per square millimeter [17]. External stimuli induce reflex tear production, blinking, and the release of trophic factors in response to dust, pathogens, and environmental stress [18]. Corneal injury resulting from infection, transplantation, autoimmune conditions, and other pathologies can lead to loss of transparency, the abnormal growth of vessels, and loss of vision [19]. Due to its external localization, environmental conditions, such as UV radiation, cigarette smoke, and vapors, are involved in OS in the cornea and in shifting the balance between oxidant/antioxidant statuses, being one of the mechanisms that contribute to cell death [20, 21].

Cornea requires robust antioxidant defenses. It expresses the three major mammalian SOD enzymes; CuZnSOD (SOD1), MnSOD (SOD2), and extracellular SOD (EC-SOD or SOD3) have been identified in the corneal epithelium, stroma, and endothelium as well as heme oxygenase-1 (HO-1) and NADPH cytochrome P450 reductase activities.

It also becomes more prone to infections, mainly due to increased epithelial permeability and impaired barrier function secondary to the focal loss of hemidesmosomes that occurs with age [16–19], as well as decreased phagocytic ability of neutrophils. These changes can be observed in lattice corneal dystrophy an oxidative stress-related disorder) associated with familial systemic amyloidosis (Meretoja syndrome). The last is caused by an autosomal dominant mutation in gelsolin gene resulting in unstable protein fragments and amyloid deposition in various organs. In the eye, ocular surface and corneal amyloid deposits were positive for the amyloid P-component protein but negative for the nonimmunoglobulin amyloid A protein and prealbumin [17–22].

For lattice corneal disruption, it has been reported that oxidant compounds such as *tert*-butyl hydroperoxide (tBHP) make corneal cells more susceptible to oxidative DNA damage and oxidative stress-induced apoptosis *in vitro* [23–25]. Besides, some authors suggest that the oxidant–antioxidant imbalance and accumulation of oxidized DNA lesions in FECD endothelium could be a consequence of the downregulation of peroxiredoxins, thioredoxin reductase, superoxide dismutase isoforms, and metallothionein without compensatory upregulation of catalase and glutathione-dependent antioxidants [26].

Then, it is well known that the presence of free radicals and proinflammatory factors induce a greater deposition of amyloids such as amyloid A, then we suggest that these factors are exacerbating the amyloidogenic process observed in these ocular pathologies.

Corneal inflammation or keratitis is characterized by tearing, blurred vision, eye irritation, pain, and corneal neovascularization altering the integrity of the cornea [26]. Infections are the main cause for the development of corneal inflammation; however, physical and chemical injury also cause it [27]. During an inflammatory process, molecules such as cytokines, growth factors, and chemokines induce recruitment of neutrophils and fibroblast proliferation causing a decrease of transparency [28]. If the physiological conditions are restored, tissue remodeling occurs in addition to vascular regression and repair of corneal epithelial cells [29, 30].

Another different amyloidosis has been identified in the cornea, such as gelatinous drop-like corneal dystrophy (characterized by subepithelial and stromal amyloid deposits), and hereditary gelsolin amyloidosis (characterized by corneal lattice dystrophy, and cutis laxa) [10]. Although several different types of amyloid fibril deposits have been observed, the main precursors identified in these two pathologies were lactoferrin, gelsolin (an 83-kDa actin-modulating protein) and light chain k protein (an Ig family member) [31]. It has been proposed that oxidized lipids could accelerate the amyloidogenesis of the 8 kDa gelsolin fragment; however, the specific mechanisms remain to be determined [31].

Finally, it is totally necessary to mention that Gelsolin has anti-inflammatory properties by inhibiting the inflammatory enzyme myeloperoxidase's activity [32] as well as antiamyloidogenic and antioxidant properties [33].

Specifically, the antioxidant function is because intracellular gelsolin has five free thiol groups (cysteinyl groups) that can engage in the oxidation/reduction reaction, meanwhile its antiamyloidogenic properties were demonstrated when gelsolin inhibited the fibril formation of the *β*-pleated sheet structure and fibril formation of amyloid *β* (A*β*) peptides in an animal model of Alzheimer's disease (AD) [33]. However, if gelsolin preserves these protective properties in gelatinous drop-like corneal dystrophy and hereditary gelsolin, amyloidosis in the eye has not been proved yet.

In addition, there is another type of amyloidosis process in the cornea. A*β* accumulation in the brain is a characteristic of AD; however, this peptide also has been detected in the different anatomical regions of the eye of AD patients and AD-mice models [34]. A*β* peptide cytotoxicity is mediated by free radical damage, and next, we present some probes previously reported: increases H_2_ O_2_ in cells in culture; this peptide also produces H_2_O_2_ through copper or iron reduction with concomitant TBARS formation; catalase, an enzyme that converts H_2_O_2_ to O_2_ and H_2_O, blocks A*β* toxicity; the presence of senile plaques induce a dramatic increase in intracellular ROS; the enzyme SOD prevented endothelial damage induced by high micromolar concentration of A*β*, suggesting that O_2_^_^ may also be playing a role in Ab toxicity; amyloid fibrils reduce copper suggesting that oxygen radical species can be generated both during the initial and late step of amyloid formation [35].

The A*β* peptide also induces lipoperoxidation of membranes and lipid peroxidation products that are involved in modifications of proteins by covalent binding. 4 hydroxynonenal (4-HNE) is an aldehydic product of membrane lipid peroxidation. The oxidative damage of proteins generates an increase in carbonyl groups due to oxidation of sensitive amino acids such as histidine, proline, arginine, and lysine. The evidence indicates that carbonyl residues and protein nitration in AD brain may come from the reaction of peroxynitrite, a powerful oxidant produced from the reaction of O_2_^_^ and nitric oxide (NO) with proteins.

Finally, the RNA and DNA are also susceptible of oxidative modification, rendering hydroxylated products of its bases. There is evidence of increased oxidative damage to cytoplasmic RNA nuclear and mitochondrial DNA in AD [35–37]. The relation between OS, inflammation, and amyloidosis in the central nervous system has been widely described in AD [38–42].

Considerable visual impairment has been reported in AD patients whereas other studies have focused on exploring potential ocular markers of AD [34, 43, 44]. The pathological accumulation of A*β* in the eye induces an imbalance in redox equilibrium, an enhanced activation of inflammation and more accumulation of this peptide. Altogether, it is a vicious circle affecting the normal functioning of corneal cells for the mechanisms previously described [34, 43–45]. A recent study found amyloid beta deposition in a transgenic model of AD (transTgAPPswePS1 mice express the human APP with the Swedish mutations at the *β*-secretase cleavage site PS1 with increased amyloid beta levels) with a diffuse pattern in the epithelial cells of the cornea as compared to controls, causing cornea epithelial cells degeneration, associated with apoptosis in basal lamina cell [44, 45]. Other studies describe the presence of amyloid-beta in the anterior segment of the eye. The external position of the anterior segment makes it easier to get biopsies facilitating its study [46–49].

Antioxidants are an alternative treatment for amyloid disorders; epidemiological studies have shown that they alleviate OS and inflammation in chronic diseases [50, 51]. Antioxidants may act as natural free radical scavengers and decrease the release of different cytokines [52, 53] (Figure 1). Antioxidant therapy has recently gained relevance in ocular diseases as potential therapeutic targets. Higher dietary consumption is associated with a risk reduction of age-related macular degeneration, cataract, and glaucoma [54–56]. Antioxidants have been tested to treat corneal neovascularization, ultraviolet B irradiation-induced damage, and Fuchs endothelial corneal dystrophy, with results that show increased corneal endothelial survival and a decrease in amyloid deposit formation [57, 58]. Although there are few studies about the role of OS in corneal amyloidosis disease, the importance of ROS and proinflammatory cytokines in the development of these pathologies is clear. Therefore, it is necessary to explore new antioxidant therapies for the treatment of corneal disease focused on decreasing the inflammatory response and amyloid aggregates formation.

As we explained before, A*β* deposition is toxic for nearby cells, induce OS, enhanced immune responses, and altogether creates a vicious circle that could be interrupted by antioxidant molecules. These could be useful for the other amyloidosis presented before in order to restore visual functions.

There are other pathologies that could affect directly the eye and offers an example that this interplay between the OS and inflammation induces secondary amyloid deposition in Alkaptonuria (AKU). Millucci et al. demonstrated that in AKU, a disease was developed from the lack of homogentisic acid oxidase activity, causing homogentisic acid (HGA) accumulation that produces an HGA-melanin ochronotic pigment and secondary amyloid-A (AA) amyloidosis. The latest is a serious complication of chronic inflammatory conditions. In AKU, the chronic accumulation of HGA and its autooxidized derivatives may initiate a variety of reactions that promote inflammatory responses and mediate tissue damage as well as repeated oxidative insults to selected target tissues initiated by HGA autooxidation.

Since 1959 has been reported histological findings on an eyeball from a patient with AKU. Hereditary ochronosis is defined as an inbred disorder of phenylalanine and tyrosine intermediary metabolism, in which the initial expression is usually the spontaneous urinary excretion of HGA. Ochronosis and alkaptonuria were at one time considered to be synonymous terms, but now it is generally agreed that the alkaptonuria or urinary excretion of HGA is only one symptom of ochronosis. In this study, it described the deposition of the melanin-like substance in the collagen bundles of the sclera and cornea and episcleral and in the elastic tissue of the conjunctiva. Now, we know that there is also secondary amyloid-(AA) amyloidosis [39–41].

### 3.2. Trabecular Meshwork

The trabecular meshwork (TM) of the eye, composed of cells and matrix, is thought to regulate aqueous humor outflow to control intraocular pressure (IOP). The TM is a porous structure that encircles the circumference of the anterior chamber of the eye [59, 60]. Its inner face borders the anterior chamber, and the outside lies against the corneal stroma, sclera, and Schlemm's canal. The TM consists of connective tissue surrounded by endothelium and consists of two parts: the nonfiltering portion, occupied by trabecular cells, and the filtering portion [61, 62]. Trabecular cells are aggressive phagocytic cells responsible for removing cell debris and proteins from the aqueous humor. The filtering portion is composed by the cribriform layer, the corneoscleral meshwork, and the uveal meshwork [61, 62].

The TM and related outflow pathways remove the aqueous humor from the anterior chamber of the eye. The intraocular pressure depends on the rate of fluid removal by the TM [61, 63]. In normal conditions, the rate of fluid removal matches the rate of its formation, but when the TM cannot keep up with the rate of fluid formation the intraocular pressure becomes elevated, which is a major risk for glaucoma, oxidative damage can result even in specific molecular changes that contribute to the development of age-related sight-threatening diseases, such as glaucoma and cataracts [3, 62, 63].

Glaucoma is a degenerative disease that affects the anterior and posterior segments of the eye. The extended damage caused by this disease is detected in the TM as biological aging, which actively participates in the development of neurodegenerative diseases. Several pathophysiological age-related changes are identified, one of them is that the TM becomes more pigmented [64, 65]. Another age-related change is the reduction of the outflow facility and the increase of the outflow that is even more evident in patients with glaucoma, this may lead to an increase in the cribriform tissue [66, 67]. This is a result of the accumulation of extracellular matrix material in the TM throughout time. The extracellular matrix material that accumulates is composed by glycosaminoglycans and proteoglycans, myocilin, thrombospondin, fibronectin, and colchicine [10, 60]. Moreover, the properties of the extracellular materials change with an increase in the deposition of electron-dense plaques [61]. Extracellular matrix deposition, OS, and TGF-*β* signaling pathways are certainly important components of glaucoma pathogenesis, and altogether can induce cell death, extracellular matrix production, and accelerated senescence [2]. TGF-*β* is a super-family of signaling proteins that have also been found at high levels in the aqueous humor of glaucomatous eyes. It is believed that high levels of TGF-*β* contribute to the progression of primary open-angle glaucoma (POAG). TGF-*β* can exist in active and latent forms, but it has been shown that thrombospondin-1, a TGF-*β* activator, is upregulated in glaucomatous tissues and may initiate the signaling cascade [68]. All these changes can be found in several age-related diseases, but also in diseases of the human eye, such as age-related macular degeneration and POAG [3]. However, the specific reasons for all these changes are still not clear. Here, we will focus on one factor that might play a critical role, i.e., OS [69]. Twenty years ago, it was hypothesized that local OS is a determining factor in the pathogenesis of glaucoma. It has been suggested that the progressive loss of TM cells in glaucoma patients could be related to the long-term effects of oxidative damage induced by free radicals. It was shown that the system called arginine/nitric oxide system is damaged in patients with glaucoma. Additionally, increased concentration of superoxide dismutase, malondialdehyde and glutathione peroxidase has been detected in aqueous humor of patients with primary open-angle glaucoma (POAG) and the activity of antioxidant system decreased in POAG [70, 71] (Figure 1).

An age-dependent increase in the production of free radicals, or a decrease in cellular repair or degradation mechanisms, will increase OS that results in the accumulation of oxidized proteins and the formation of amyloid. Chronic OS can contribute to reduced outflow by inhibiting the intracellular proteasome system that works to degrade intra- and intercellular debris. After exposure, oxidative conditions induce a loose proteasome activity and have increased cell death [64]. The accumulation of the latter can lead to oxidative insults and mitochondrial dysfunction. Thus, the oxidative damage related with aging could be the main cause of the functional decline of mitochondria, promoting decreased ATP production, impaired oxidative phosphorylation system, and reduction in the number of mitochondria [64].

The human TM is the most sensitive tissue of the anterior chamber to oxidative damage as the result of not being directly exposed to light. The consequence of being hidden in the sclerocorneal angle is a reduced antioxidant defense [72]. ROS (induced by light) change the oxidant-antioxidant balance in the aqueous humor. The aqueous humor is known for containing several oxidative agents like hydrogen peroxide and superoxide anion [73]. This OS stimulates the antioxidant enzymatic system and decreases the antioxidant potential in the aqueous humor; therefore, with the increase of ROS, the levels and activity of protective superoxide dismutase and glutathione peroxidase decreased resulting in the oxidant-antioxidant imbalance [72]. It has been suggested that chronic oxidative conditions, resulting from this oxidant-antioxidant imbalance, may compromise TM function [73]. Oxidative stress to the TM can cause several damages such as reduced TM mitochondria respiratory activity, growth arrest, alteration of extracellular matrix composition, structure and accumulation, damaged TM cellular DNA, altered membrane permeability, rearrangement of TM cellular cytoskeleton, loss of cell-matrix adhesion, altered cell cycle progression, inflammatory cytokine release, apoptosis, as well as many forms of cell death [74]. Cell death may cause free radical production that results in the loss of TM cells, which leads to an increase in OS promoting the beginning of a vicious cycle [73]. It is assumed that TM cell loss is caused by OS-induced apoptosis via inflammation, mitochondrial damage, hypoxia, and endothelial dysfunction [69].

Oxidative status affecting the TM cells may promote oxidation of their DNA, the oxidative DNA damage in TM correlates with glaucomatous elevated mean intraocular pressure and visual field damage [75]. In the aqueous humor of patients with open-angle glaucoma, the antioxidative capacity was found to be reduced by more than 50% compared with nonglaucomatous eyes [73]. Furthermore, an increase in 8-hydroxy-2′-deoxyguanosine (Figure 1), a molecular biomarker for oxidative damage, was detected in the TM samples of glaucoma patients who underwent standard filtration surgery [69]. Patients with open-angle glaucoma have a higher susceptibility to oxidative damage since their antioxidant potential is reduced by 60 to 70%, although the activity of antioxidant enzymes is increased in the same amount [72]. The TM cells are in contact with high concentrations of oxidative agents such as hydrogen peroxide. This exposure does not affect the outflow in the normal eye; however, it causes a 33% decrease in outflow in reduced glutathione-depleted eyes [75]. Open-angle glaucoma patients display a genetic background that makes them susceptible to ROS-induced damage, since the GSTM 1-null genotype, a deletion of the gene encoding glutathione S-transferase, has been found to be significantly more common in these patients [76].

Recent reports have demonstrated, in patients with POAG, the occurrence of mutations in the mitochondrial genome and, as a result of these mutations, a reduced mitochondrial respiratory activity in comparison to control subjects [73]. ROS attack DNA by purine or pyridine bases structure changes. Yet, protein cross-linking and breaks in the DNA string were detected. These mutations can concern nuclear as well as mitochondrial DNA (mtDNA). If the mitochondrial DNA is affected, resulting in impaired energy production. Additionally, OS seems to have a key in the pathogenesis of glaucomatous nerve atrophy. Damage of DNA, caused by free radicals, correlates significantly with IOP as well as wit visual field defects and could be shown in TM cells in glaucoma patients. Furthermore, an increased lipid peroxidation, being the result of ROS-induced cell damage, was noticed as well as an impairment of metalloproteinases-2 (MMP-2) expression, resulting in an accumulation of extracellular matrix. This accumulation (i.e., plaques) in TM could be seen several years before by electron microscopy studies [77, 78].

Free radicals are considered the triggering agents in cellular decay; hydrogen peroxide exposure alters the adhesion of TM cells to extracellular matrix proteins, which results in rearrangements of the cytoskeletal structures that may induce a decrease in TM cell adhesion, cell loss, and compromise TM cells integrity [79]. TM cellular declines linearly with aging; however, it is more severe in glaucomatous patients in comparison with nonglaucomatous subjects of the same age [80]. The precise mechanisms that contribute to TM cell loss are not known yet; however, it has been proposed that the combination of a higher level of hydrogen peroxide with insufficient glutathione levels induces collagen matrix remodeling and TM cell apoptosis [72]. These OS-induced changes were reduced by pretreatment with prostaglandin analogues [68, 81].

There is evidence that supports that age-related reduction of the outflow facility represents the equivalent to systemic arteriosclerosis [65]. Low-grade chronic stressors, such as oxidation, may result in inflammation. Continuous exposure of TM cells to OS, via hydrogen peroxide, results in ROS generation. This, in turn, stimulates NF-*κ*B, which regulates the expression of genes involved in inflammation, oxidative stress, and endothelial dysfunction [80, 82]. Thus, NF-*κ*B lies at the crossroad between OS and inflammation. ROS activates the NF-*κ*B, which induces the expression of several genes, including the expression of the inflammatory cytokines IL-1 alpha, IL-1 beta, and IL-6. IL-1 produced by the TM inhibits apoptosis but can cause accumulative damage associated with oxygen free radicals' production [72]. The positive feedback loop between NF-*κ*B activation and IL-1 expression could be a response to a lower intraocular pressure. This supports the hypothesis that the activation of the inflammatory pathway is a cellular attempt to decrease intraocular pressure [81]. IL-1 lowers the intraocular pressure possibly by the stimulation of matrix metalloproteinase (MMP) expression, or by increasing paracellular permeability across the Schlemm's canal [74]. In the eye, IL-1 stimulates increased levels of MMP-3, MMP-9, and MMP-12 that are essential in maintaining intraocular pressure homeostasis by influencing extracellular matrix turnover. IL-1-induced MMP-3 increases the aqueous outflow regulating the permeability of the Schlemm's canal [74].

### 3.3. Trabecular Meshwork and Amyloidosis

We already have mentioned the Transthyretin (TTR)-related familial amyloidotic polyneuropathy (FAP) and its consequences in the cornea. This pathology also affects the trabecular meshwork. TTR is a circulating protein forming a stable tetramer, synthesized in the liver and the brain's choroid plexus, the retinal pigment epithelium (RPE), and the small intestine. It acts as a thyroxine and vitamin A transporter, and it carries retinol and in a lesser amount, thyroxine (T4) when mutated, its tetramer conformation turns into monomers that aggregate and form amyloid deposits [75, 76]. The most serious ocular manifestation of FAP is glaucoma [76–78], and it has been suggested that the loss of peripheral nerve fibers in FAP, and the consequent ischemia, is caused by endoneurial amyloid deposits and the toxicity of amyloid deposits to TM cells [75].

An ultrastructural study of the TM of this patient showed the presence of extensive amyloid deposits in the intertrabecular spaces and the Schlemm's canal. TM's deposition of amyloid obstructs the aqueous humor outflow and subsequently elevates intraocular pressure [79]. The pathophysiological mechanisms responsible for the elevation of the intraocular pressure include perivascular amyloid deposition in conjunctival and sclera tissues, intratrabecular deposition, and deposition of amyloid on the pupillary edge [75].

Amyloid deposition in the vitreous humor causes a subsequent gradual decrease in visual acuity, this is a characteristic throughout the natural course of the disease. The main ocular manifestation present in FAP patients include vitreous opacities, chronic open-angle glaucoma, abnormal conjunctival vessels, keratoconjunctivitis sicca, anterior capsule opacity of the lens, retinal vascular changes, and optical neuropathy [75]. Due to the local production of amyloid fibrils, vitreous opacities may recur after vitrectomy, inducing open-angle glaucoma development. Vitrectomy increases oxidative conditions in the TM and, in the absence of the vitreous, the amyloid aggregates reach the TM and Schlemm's canal more easily. This suggests that the vitreous acts like a filter that retains the amyloid fibrils and prevents their progression to the TM [75–79]. In spite of all these studies identifying amyloid deposition as a risk factor for FAP, there is no more information about the molecular and biochemical mechanisms participating in the development and progression of this disease. More studies are needed in order to know if a direct interrelationship between the three factors: OS, inflammation, and amyloidosis are involved in glaucoma. Based on what we have explained before, we could suggest that the toxic feedback loop is present in TM and physicians, and pharmaceutic companies should take in count the design of treatments or multitarget therapies.

### 3.4. Aqueous Humor

While most of the research about Alzheimer's disease (AD) and the anterior segment (AC) of the eye has focused on the crystalline lens, little is known about the potential presence of amyloid biomarkers in the aqueous humor (AH). The AC is considered a highly specialized vascular compartment whose inner walls are composed of the iris, cornea, and trabecular meshwork endothelia [80]. The AC encloses the aqueous humor (AH). The volume of the AC is 0.25 mL, whereas the volume of the posterior chamber is 0.06 mL approximately. The AH is necessary to guarantee optical transparency, structural integrity, and nutrition of AC structures [81]. Moreover, the AH has the function of protecting and supplying nutrients to the cornea, lens, and TM [68, 82]. Other functions ascribed to AH inflow have been less clearly defined [83] and include the delivery of antioxidants and participation in local immune responses. The AH is a fluid with an ionic composition very similar to the blood plasma and the cerebrospinal fluid (CSF). It has been proposed that A*β*40 and A*β*42 plasma concentrations correlate with a higher risk of AD [84, 85]. Nevertheless, other studies hold the opposite and the presence of these peptides in AH has been previously reported [86–88].

Anterior ocular fluid and CSF share a number of features, such as the similarity between blood–aqueous and blood–CSF barriers [89]; both are modified extracellular fluids responsible primarily for nurturing several specialized types of cells in the eye and in the brain, respectively, in addition to performing an important hydromechanical function [90]. They also have a common embryological origin, arising from neuroectoderm tissue. Some authors postulate the eye as the fourth brain ventricle, and, therefore, it is possible to find amyloid peptides in the AH [91].

To our knowledge, the only study that analyzed the HA in AD individuals was published by Tripathi et al. They reported the presence of tau protein in both the CFS and the HA [89]. A recent meta-analysis reported that core CSF biomarkers of neurodegeneration (T-tau, P-tau, and A*β*42) and plasma T-tau were strongly associated with AD [92]. Goldstein et al. identified A*β*40 in the human aqueous humor that is comparable with those identified in aged human CSF, using anti-A*β* mass spectrometry analysis of aqueous humor samples of three AD-free individuals undergoing cataract extraction [93]. On the other hand, Prakasam et al. reported significant amounts of A*β*40 and A*β*42 in the AH of bovines and transgenic mice through ELISA testing and in aqueous humor from 255 human cataract patients [86, 94].

The A*β* peptide and AD-related protein levels have been described in the AH of patients with pseudoexfoliation syndrome (PEX) and glaucoma; thus, lending themselves to being related with neurodegenerative etiologies [95, 96]. Janciauskiene et al. studied a sample of aqueous humor specimens obtained during cataract surgery in patients with cataracts only or combined with glaucoma, PEX, macular degeneration, and diabetic retinopathy. They described measurable levels of A*β*38, A*β*40, and A*β*42 in at least 40% of all samples, except for the diabetic retinopathy group for which A*β*38 was identified in only 31% of cases [97]. On the other hand, Lesiewska et al. were unable to prove any relation between PEX and Alzheimer's amyloids or cognitive functions in cataract patients [98]. Taking these results into account, it would be interesting to examine A*β* and Tau proteins in the AH of individuals with neurodegenerative disorders, which might reveal higher levels of some of them, as they are found in neocortical deposits.

### 3.5. Lens

The lens is a nearly transparent biconvex structure suspended behind the iris of the eye and focus light rays onto the retina. The lens is made up of elongated cells that have no blood supply, but it takes nutrients mainly from the aqueous humor.

Cataracts are the most common cause of vision loss in aged people. Cataract formation includes the deposition of aggregated proteins in the lens and damage to the plasmatic membrane of lens fiber cells [2].

One of the primary changes during aging is the increase of the relative thickness of the lens' cortex throughout a person' s life. This change increases the curvature and, therefore, the refractive power of the lens, with the concomitant deposition of insoluble particles that, at the same time, decrease the refractive index. Therefore, the eye may become more hyperopic or more myopic in aging [2, 99].

Chaperones contribute to ensure quality control mechanisms to achieve an adequate protein function under normal and stress circumstances [100]. Mitochondria contain two particular chaperones: human heat shock proteins (Hsp) 60 and 70, which protect damaged proteins in the aged eye [4]. The Hsp alpha-crystalline is made of two polypeptides, alpha A crystalline and alpha B crystalline, these are the predominant proteins of the eye lens in vertebrate animals. Alpha A is key for lens transparency, ensuring that alpha B or other close related proteins remain soluble [1, 101].

Nevertheless, as the cell fibers of the lens grow, a proteolytic cleavage of crystalline induces a gradual conversion from water-soluble into water-insoluble proteins. These changes induce aggregation that, in turn, provokes subsequent light scattering and lens opacity. Complement components contribute to pathogenic processes by damaging tissues, increasing chemotaxis, and facilitating neovascularization [102]. Montalvo et al. established an association between C1q, C3, C4, and corneal and lens anterior capsule damage without inflammation [103], suggesting that molecules released by inflammatory cells and inflamed tissues may affect adjacent tissues not directly involved in the pathogenic process [1, 104].

Similar to other basal membranes (BM), the lens capsule, a BM secreted by the lens epithelial cells, tends to accumulate posttranslational modifications in aging, since the proteins that constitute BMs usually present a low turnover rate [105]. Raghavan et al. reported advanced glycation end products (AGE) accumulation in the human lens capsule with increasing age, which in turn is associated with a higher incidence of cataracts [106]. In addition, recent studies suggest that AGEs bind to a cell surface receptor known as RAGE. RAGE belongs to the immunoglobulin family of receptors [107]. AGE-RAGE interaction increases intracellular OS by activation of the NADPH-oxidase, a key mediator in superoxide radical production [107]. Therefore, AGEs are linked to another hallmark of aging: OS.

Studies have shown that SOD2 is helpful to protect lens epithelial cells, since cells with a high-level expression of this enzyme show resistance to the cytotoxic effects of H_2_O_2_, O^2-^, and UVB radiation [108]. On the contrary, SOD2-deficient cells show mitochondrial damage, leakage of cytochrome C, caspase 3 activation, and increased apoptosis when exposed to O^2-^ [109]. Based on these results, one could conclude that this system is of great significance to ocular and general health maintenance. Therefore, as its efficacy decreases with age, several eye diseases may develop.

### 3.6. ROS in the Lens

As previously mentioned, ROS are generated from intrinsic and extrinsic sources. Through the years, the lens becomes a tissue highly susceptible to oxidative damage since the proteins that constitute it are never replaced. Consequently, protein oxidation, DNA damage, and lipid peroxidation are all found in the process of cataractogenesis [110]. Nevertheless, since the lens has a high concentration of reduced glutathione, as previously mentioned, it helps to maintain reduced thiol groups, leading to transparency of the lens and cornea, and concentration of nuclear glutathione (GSH) helps to prevent oxidation [111].

The lens is a part of the eye less studied in terms of the expression and function of amyloid precursor protein (APP) and its cleavage products such as A*β* peptide. APP and the proteolytic enzymes involved in A*β* production were found to be expressed in the lens and in the vitreous fluid [112].

It has been described that A*β* 1–42 and A*β* 1–40 at low nanomolar concentrations reduced oxidative damage in human lens epithelial cells (HLECs). These cells were treated with H_2_O_2_ as an OS model *in vitro* and both beta amyloid peptides shown antioxidant effects in a concentration-dependent manner. These properties have been described previously in neurons and in lipoproteins of cerebrospinal fluid [113]. The proposed antioxidant mechanism explains that A*β* peptides are regulators of *insulin-like growth factor binding protein-3/5* (*IGFBP3/5*) gene expression which has a known antioxidative effect enhancing the expression of antioxidant enzymes such as Mn-superoxide dismutase, Cu/Zn superoxide dismutase and glutathione peroxidase-1 [112, 113].

On the contrary, it is well known that all the toxic effects of A*β* peptides and in these cells at micromolar concentrations induce the activation of apoptotic processes, mitochondrial damages, the overproduction of ROS and proinflammatory responses [114]. Then, we can conclude that the three factors: OS, inflammation, and amyloidosis are in a constant interplay affecting the normal functions of lens cells.

## 4. Conclusions

Changes in the anterior segment of the eye are responsible for half of the four most common causes of age-related vision-impairing diseases (glaucoma, cataracts, age-related macular degeneration, and diabetic retinopathy). The burden of these ocular diseases will not only affect developed countries but also developing regions with limited resources. The structural and molecular changes observed in the anterior eye segment are caused by molecular changes in intercellular unions, structural arrangements of collagen fibers, overexpression of degradation enzymes, underexpression of inhibitors of metalloproteases in tissues, absorbed UV light that produces ROS, inflammatory cytokines among others. These changes are activated by an imbalance and interplay of OS state, inflammation, and amyloidosis, altogether cause an abnormal functioning of the anterior segment (Figure 1).

As the individuals age, this interplay is greater evolving into a vicious circle that will ultimately increase the development and progression of ocular pathologies that can cause vision loss. Multifactorial therapies should be developed to stop the progression of ocular pathologies and vision loss in aged patients but more importantly, changes in lifestyle are needed in order to avoid the chronic oxidant and inflammatory state responsible of protein misfolding. It has been recognized that the anterior chamber of the eye permits the survival of foreign tissue and tumor grafts. There are anatomical and physiological characteristics, as well as dynamic immunoregulatory factors that contribute to ocular immune privilege. It is necessary to mention that ocular fluids contain several immunosuppressive and immunoregulatory factors that suppress T-cell proliferation and secretion of proinflammatory cytokines. Fas ligand (CD95L) is present in the interior of the eye, and this factor is responsible of the apoptosis of infiltrating inflammatory cells.

The entry of antigens into the eye induces a specific immune deviation in which TH1 responses are suppressed. These inflammatory responses reduced are an important adaptation for preventing immune-mediated injury to ocular tissues that have little or null capacity to regenerate and it allows the preservation of vision [115].

**Figure 2:**
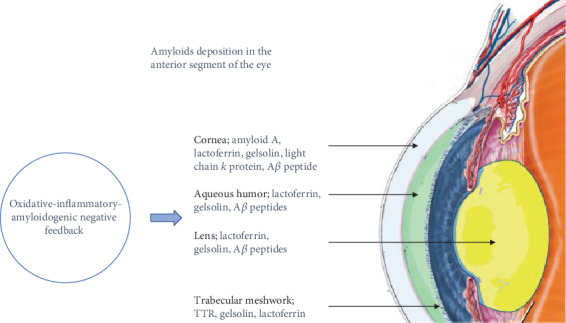
Effect of oxidative-inflammatory-amyloidogenic negative feedback in the amyloid deposition in the anterior segment of the eye. The negative feedback induces amyloidosis in the cornea, aqueous humor, lens, and trabecular meshwork.

The intracellular and extracellular deposition of circulating proteins cause systematic amyloidosis (Figure 2). TTR amyloidosis and the accumulation of A*β* peptide in the eye could be considered as antigens; however, the interior of the eye is an immunoprivileged site.

An emerging therapy for ocular amyloidosis and other amyloidogenic processes in different tissues is the use of immunotherapy (Figure 1) [116]. Immunotherapy is the treatment of disease by activating or suppressing the immune system. There are two types of immunotherapies called active or passive immunotherapies; the first is directed to amplify the immune response and the second to reduce or suppress the immune response. Immunotherapy for TTR amyloidosis has been broadly studied. Three options have been developed to reduce TTR deposition and clearing amyloid deposition; a peptide called TTR115-124, the monoclonal antibody MAb39-44, and a TTR mutant (TTR Tyr78Phe). These immunotherapies were successful to recognize circulating TTR and reduce TTR amyloidosis *in vitro*. However, they did not show if these options reduced TTR amyloidosis in the eye in vivo [117–120].

Amyloid-beta deposition has been an attractive target for immunotherapy in AD [121]. Encouraging results were obtained after the administration of anti-A*β* antibodies in mouse models of age-related macular degeneration (AMD) and AD that motivated human clinical trials; however, the first-generation of A*β* vaccines in AD was interrupted because an increase in microhemorrhage and did not remove plaques [112, 121]. Specifically, the treatment with an anti-A*β* antibody intraperitoneally in a mouse model of AMD yielded a decrease in A*β* deposits both in the retina and the brain [112, 122]. To the best of our knowledge, there are no available studies in which molecules used for immunotherapy are administered directly to the eye. As a final statement, immunotherapy studies may provide promising diagnostic, safety, and therapeutic tools, targeting pathological amyloidosis the eye in the future. However, immunotherapy directed for other pathologies such as metastasic melanoma can cause a vision-robbing side effect [123]. Effective immunotherapy for chronic infectious diseases will require the use of appropriate target antigens; the optimization of the interaction between the antigenic peptide, the antigen-presenting cell (APC), and the T cell; and the simultaneous blockade of negative regulatory mechanisms that impede immunotherapeutic effects.
